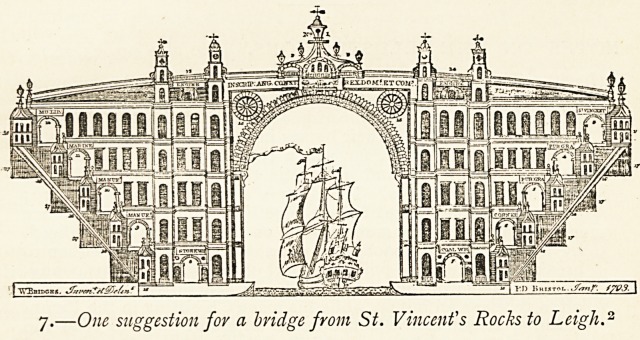# The Reputation of the Hotwells (Bristol) as a Health-Resort

**Published:** 1902-06

**Authors:** L. M. Griffiths


### THE REPUTATION OF THE HOTWELLS (BRISTOL) AS A HEALTH-RESORT.

BY

L. M. Griffiths, M.R.C.S. Eng.

*(Continued from p. 26.)*

---

In 1793 Dr. John Nott, practising in Dowry Square,[1] compiled a little treatise for the use of the various visitants at the Hotwells, which reached a third edition after 1802, but he does not "dwell on analytical minutiæ, which are alone interesting to the chymist." Nott devotes a section of his pamphlet to the hot spring which had been newly discovered on Clifton Hill. The water, raised by a fire-engine, proved convenient to the invalids who were beginning to occupy the residences provided for them at the top of the hill.[2] Nott

[1] The *Bristol Directory* of 1799–80 gives his address as 1 Chapel Row, which would be considered part of Dowry Square. It was the house occupied by Elizabeth Trinder in 1754 (see page 15, and *Bristol Directory*, 1809).

[2] Lady Hesketh, cousin of the poet Cowper, wrote, on September 16, 1799, concerning "this most charming place Clifton Hill, which is just now in high beauty ; The Woods which Crown these charming Rocks being as green as in June, and the Verdure of the whole country *Intense!* I think you would be greatly charmed and delighted could you see the sweet sublime yet peaceful Views, which I enjoy from every window in the house, for tho the Bristol people have done all in their power to ruin the Rural beauty's of Clifton Hill by the number of abominable Buildings they have erected all over it, yet *Nature* has been so profuse of her bountys in the disposition of the Ground and the happy Combination of wood, water, Rocks, &c. that it is always preferable to any other place." (*Letters of Lady Hesketh to the Rev. John Johnson, LL.D.*, 1901, p. 91). When Lady Hesketh was here in 1796 she found that "the Sweet and pure air of this Enchanting Spot had greatly taken off that constant Hectic fever which had hung upon her so long" (p. 50).

came to the conclusion that the water came from the spring that supplied the old wells. In relation to the present campaign against tuberculosis, it is interesting to learn from Nott that "how far consumption is catching agitates the mind of many a friend attendant on the unfortunate. As I do not consider the matter of suppurated lungs as a specific contagion, so I think it cannot positively be communicated. Whether the air of a room be contaminated with the purulent effluvia of pulmonic ulceration, or of a suppurating sore of any other part of the body, it will be equally injurious to the health of attendants; and as such attendants are most often relatives of the sick, and possibly predisposed by inheritance to the same disease, so they more readily than others may have their lungs affected by the effluvia of matter. Perhaps also matter breathed from the lungs may be more largely and intimately diffused through the air, than if it exhaled from an open ulcer, and was simply absorbed; hence it affects with greater facility. Were the matter of consumption a specific poison *sui generis*, a mortality must frequently prevail amongst our Hotwell nurses."[1] Nott considered that the utility of the Bristol water in phthisis was owing to the fact that "the terrene matter corrects all acidities of the primæ viæ ; it absorbs all acrimonious humours of the habit; prevents their accumulation, and erosion of the blood-vessels, so as to create hæmorrhage; in which their native stypticity has also its effect ; it involves the saline particles, enabling them to pass through the larger tubes of the body without effect, till they arrive at the smaller canals, where this terrene matter, unable from its grossness to pass, quits them ; and the salts then act with their destined efficacy," and that therefore the water "prevents, or even resolves those scrofulous obstructions of the infinitely minute glands of the mucous membrane of the lungs, which possibly constitute tubercles. It corrects the septic matter of their suppurations, and carries it innoxious out of the constitution. It prevents the hæmorrhage, when the sanguiferous system of the lungs is eroded from matter, or when ruptured from an increase of circulation there determined. And lastly it restrains the night sweats, and

[1] *Of the Hotwell Waters, near Bristol*, 1793, pp. 73-4.

mitigates the fever."[1] But both in phthisis and diabetes, wherein it is of approved efficacy, concomitant aids must not be neglected. The general scheme of regimen which Nott laid down as necessary in the treatment will be seen in what he describes as

THE INVALID'S DAY.

> At six in the morning take asses' milk, diluted, or otherwise. Rest about an hour after it in bed; should perspiration ensue, which is frequently the case, rest rather upon the bed, lightly clad. Rise at seven, or earlier. Be at the Wells by half past seven, there take the first glass of water; and, having walked in the open air, if the weather permit, otherwise under the colonade, for twenty or thirty minutes, take the second glass. Ride on horseback, or in a carriage, from eight to nine. Breakfast, and the private avocations of the morning will engage till —Twelve, when a customary medicine is to be taken. At one go to the waters, and drink two glasses in the same way as in the morning. From half past one ride on the downs, or elsewhere, till four. Dinner. Remain quiet after it, or perhaps repose on a couch till six, then repeat the usual medicine. Half hour after six take tea, or such habitual beverage. At seven walk, or, should debility forbid, ride. At eight, or soon after, be returned home. At nine, or soon after, make a light supper, if accustomed to such meal. At eleven take the night medicine, and retire to rest. But this distribution of hours must be varied according to the degree of the disease, and other circumstances. Many invalids cannot rise so early as six, and will wish to be in bed before eleven; neither will they bear the exhibition of the Hotwell water twice in the day.[2]

Nott is very emphatic in his praise of the loveliness of the neighbourhood, and speaks of Ashton as a village which "three miles in length, is one continued bed of strawberries." He bears testimony to the sufficiency of the amusements, but he laments "that the female invalids at the Hotwells, who are for the most part at that period of life when public entertainments have their peculiar relish, err in no one instance so much as in the indulgence of dancing; an exercise most salutary to lungs that are sound, but as injurious to those that are unsound."

Dr. Thomas Beddoes, having given up his chemical lecture-

[1] Pp. 64-5. [2] Third Edition, pp. 94-6.

ship at Oxford, settled in 1793 in Hope Square at the Hotwells, a neighbourhood in which, on account of its reputation with consumptive and other patients, he thought he might have exceptional facilities for putting to a practical test his views on the treatment of disease by the inhalation of "factitious airs." In the early part of that year had appeared his *Observations on the Nature and Cure of Calculus, Sea Scurvy, Consumption, &c.*, in which he had put forth the theory that there is "an excess of oxygene in the system of consumptive persons."[1] He therefore thought that in phthisis, and in many other diseases, the line of treatment should be by inhalation of modified air. In *A Letter to Erasmus Darwin, M.D.*, dated June 30th, he gave details of the treatment he proposed to base upon this theory. Of the three airs—"azotic, hydrogene, carbonic acid"—he was hoping "most from the employment of hydrogene to reduce the air of the atmosphere to a lower standard. It was inhaled through a tube, and in consequence of pressure on the reservoir, a strong current set into the mouth."[2] Beddoes records[3] minutely the results of experiments made on himself with oxygen, and on the strength of them he looked forward to the time when an apparatus for its use would be "ranked among the ordinary articles of household furniture."[4] Darwin, who gave Beddoes much encouragement to persevere with his investigations, took occasion, in his reply, to state his belief that consumption was "infectious to those who sleep with such patients in the last stage of the disease."[5] An important book soon followed; for in 1794 and 1795 were issued the first and second editions of *Considerations on the Medicinal Use, and on the Production of Factitious Airs*, the medical part of which was written by Beddoes, and the rest by James Watt, the great engineer,

[1] P. 114. This new theory, as a writer in the *Monthly Review* for November, 1793, pointed out, owed its origin (mainly through the work of Priestley) to pneumatic chemistry. The reviewer offers for consideration some suggestions which might modify the opinions that Beddoes held in reference to the question of a diminished proportion of oxygen being the effect of pregnancy, and on the influence of pregnancy in suspending the progress of phthisis.

[2] Pp. 44, 46. [3] P. 50. [4] P. 55.

[5] P. 64. Beddoes himself, in opposition to much of the medical opinion of the time, did not believe in its contagiousness. (*Hygëia*, vol. ii,, 1808; Essay vii., pp. 95–8.)

who devised the apparatus[1] used. In this book first appeared the proposal for a Medical Pneumatic Institution "for the benefit of the wealthy as well as of the indigent."[2] In 1795 between £800 and £900 had been subscribed[3] towards the £1,500 which Beddoes considered was the lowest amount that would justify him in beginning the work.

The three springs mentioned by Lucas were not the only candidates for public favour. In addition to the cold bath at Jacob's Wells, there was the spring which was available at the top of the hill. And towards the close of the eighteenth century, but not earlier than 1794, "A Gentleman of the Faculty" issued an undated pamphlet, entitled *An Impartial Inquiry into the Nature and Qualities of the New Saline Mineral Spa Water at the Tennis Court House, Hotwells Road, Bristol.* The house occupied the site of what is now known as Poole's Coal Wharf. The Gentleman of the Faculty proclaimed the virtues of the Spa with no uncertain sound. The spring is described as the "inimitable Chemistry of Nature," "inestimable Saline Chalybeate Water," and "this surprising Mineral Water Spring." It was said that it did "not contain the quantity of salts found in the Cheltenham Waters, consequently more efficacious in many cases, as it is more readily taken up by the absorbent vessels, and carried through the round of circulation, and enters the whole habit, without passing off so rapidly by the intestinal tube, but at the same time acting as an undeniable alterative, though in the mildest manner, even on the most irritable and delicate constitutions."[4] Not only were there the usual facilities for its internal use, but provision was made for its external application, and the Gentleman of the Faculty pointed out "that the Hot-Wells nor even the ancient City of Bristol, have never before had any regular

[1] The book contains many drawings of the apparatus. Some of these are reproduced by Mr. George M. Foy, in an article on "The Discovery of Modern Anæsthesia" in the *Dublin Journal of Medical Science* of December, 1896. Mr. Foy says a great deal about the work at the Hotwells.

[2] Part I., Second Edition, p. 15.*

[3] The names of the donors are given on pp. 111–2 of Part III.

[4] Pp. xii., xiii.

establishment of Baths."[1] But as the popularity of the Hotwells as a pleasure-resort was declining, this Spa had only a short existence. Mr. Latimer records that in 1808 "the spa with its 'pleasant garden bordering on the river' was advertised to be let, and in January, 1810, the premises were converted into the 'Mineral Spa coal wharf.'"[2]

In 1797 Dr. Andrew Carrick issued a *Dissertation on the Bristol Hotwell Water*, in which he recorded his own analysis of it. Part I. of Carrick's work deals at great length with the chemical properties of the water derived from the two springs[3] on the right bank of the river and the Sion Spring, the name given to the one on the higher level, all of which, notwithstanding some striking differences, he considered were derived from the same source. He concluded that "a wine gallon of 231 cubic inches is impregnated with—

| | | |
|---|---|---|
| Muriated magnesia ... ... ... ... | $7\frac{1}{4}$ | grains. |
| Muriated soda ... ... ... ... ... | 4 | ,, |
| Vitriolated soda [sulphate of soda]... | $11\frac{1}{4}$ | ,, |
| Vitriolated lime [sulphate of lime] ... | $11\frac{3}{4}$ | ,, |
| Carbonated lime ... ... ... ... ... | $13\frac{1}{4}$ | ,, |
| Making together of solid matter.. | $47\frac{3}{4}$ | grains |
| Carbonic acid gas ... ... ... | 30 | cubic inches |
| Respirable air ... ... ... ... | 3 | ,, |
| Making together of gaseous fluids ... ... ... ... | 33 | cubic inches."[4] |

Carrick had no doubt of the general usefulness of the water. He stated that many "Europeans returning from tropical climates would derive much more benefit from the use of this, than any of those mineral waters, in which iron, or any other

[1] Owen (*Op. cit.*, p. 159), describing the "old Hot-well," mentions "the little private baths for one person only at a time, for which they pay one shilling each time, the bath being fresh filled for each person. These are chiefly frequented by those afflicted with different kinds of weaknesses. Many persons also in health often make use of these baths, in order to cleanse and refresh themselves, which they do to admiration.'

[2] *The Annals of Bristol in the Eighteenth Century*, 1893, p. 507.

[3] Carrick changes the names of Old and New to which Lucas devotes attention.

active stimulant is an ingredient, whatever their reputation may be. For more than a century, the Hotwell-water has been celebrated as a remedy for Diabetes, comparatively a rare disease, and one of the most obstinate; and the proportion of cures performed by these waters, is highly creditable to their efficacy. But the disease for which the Hotwells are chiefly resorted to is Pulmonary Consumption. It is above a hundred years since they were first brought into notice for the cure of this disorder; and they have ever since continued to rise in reputation."[1] After giving many details for the guidance of invalids, he concluded with some general "Practical Observations on the Prevention and Treatment of Pulmonary Consumption" intended for the lay public, for whom he narrates its usual clinical course, and affords them information as to the predisposing causes. He combats the opinion held then by some that disease is not hereditary, but admits that consumption is frequently found where no hereditary predisposition can be traced. In many of these cases the origin must be sought in a full diet or strong liquors. Carrick was much vexed with the excess of wine which was common in those days. "In the higher ranks, he who drinks one bottle only, reputes himself a sober man; and he who does not exceed half that quantity daily, is considered as remarkably temperate. It were better, for young men at least so far as health only is concerned, and abstractedly from considerations of morality, to get drunk once a week, and abstain entirely the other six days, than regularly to indulge in what may be called a moderate allowance of Port, or Maderia, or other strong wines, every day. The mother, afraid as it would seem, least her darling child should retain, any portion of vulgar health, carefully initiates him into the mysteries of Bacchus, from the very cradle; and as soon, almost, as little master is capable of swallowing, he is indulged with his regular allowance of wine. No wonder that Gout, Dropsy, Schirrous Liver, &c., should make such a conspicuous figure in the history of his 'life and sufferings.'"[2] Carrick also devotes much consideration to the hygiene of education and dress. He says "From almost continual motion, arises the

[1] Pp. 68-9. [2] Pp. 103-6.

pleasure and the health of childhood: How unnatural then, to send a child of four years of age to be immured in a suffocating school-room, and chained to a bench half the day, merely to be out of harm's way. The quality of the cloathing must likewise possess considerable influence on the health. In modern Europe, a preference is universally given to linen, as an immediate covering for the skin, on account of its superior elegance and cleanliness; but in many respects it is, perhaps, better adapted to the latitude of Egypt, whence it originally came, than the cold regions of the North. Although woollen is less agreeable to the eye, and perhaps less pleasant to the skin, the difficulty with which it suffers either cold or heat to pass, gives it a decided superiority as an article of cloathing, in such a variable climate as ours. Abstractedly from ideas of taste, every thing tight in dress, by compressing the parts and cramping motion, is injurious to health. I have more than once observed a temporary spitting of blood excited by wearing the waistcoat too narrow, or by buttoning the coat over the chest. But the injury to the female sex from the use of long and strait stays *was* much more serious and extensive. The mischievous effects of this absurd and unnatural fashion on the tender frames of young women, could only be equalled by its own innate deformity, and it is to be hoped that long waists will never again disfigure the persons of our fair country women. The loose Grecian dress of the present day is not less conducive to health than to elegance; and although it is not long since the waist has escaped from bondage, a sensible improvement in health and beauty, will, I doubt not, soon be apparent."[1] Among exciting causes of consumption Carrick considered that catarrh was most frequent, and he alludes to the attempts which had been made of "establishing a chemical diagnosis" by early examination of the sputum in all cases ot expectoration. His own experiments in this direction were too capricious for any confidence to be placed in them. By attention to preliminary warnings, and by appropriate precautions, he considered that, in at least nine-tenths of the cases, threatening consumption might be warded off, and he laid it down that in

[1] Pp. 111-3, 115.

the more early stages of the confirmed as well as in the incipient consumptive the greatest dependence should rest on "the anti-phlogistic regimen, blisters, setons, and particularly the lancet," and that the objection to bleeding as likely to increase the debility was "founded rather on supposition than actual observation." Much space is devoted by Carrick to the various palliative remedies adapted to the relief of particular symptoms, and the desirability of treatment by many "factitious airs" is explained, and these are recommended if they can be used on a thoroughly scientific basis. Carrick had given a good deal of attention to the infectiousness of phthisis; but he had not given contagion as a cause, "no case having occurred to me, where it could be positively traced; and it has always appeared to me more easy and natural, to account for the supposed cases of infection upon other principles. Where several persons of a family become affected with the disorder, one after another, it is in general only a proof of a common hereditary predisposition; and even where husband and wife successively fall victims to the disease, before a proof of infection can be established, it will be necessary to take into the account the great frequency of predisposition to the disease in this country, together with the hurtful effects of fatigue, watching and confinement, which a person in such circumstances usually undergoes, and which might have been equally injurious, and equally productive of consumption in this person, had the former relative died of dropsy or any other lingering distemper. But the strongest negative proof is afforded by the nurses at the Hotwells, who, were the disease infectious, could not possibly escape; whereas I never knew any one of them affected with it."[1] It will be seen that Carrick's views about the infectiousness of phthisis agreed with those of his neighbour Nott. Carrick was careful to emphasise the warning against the practice of

[1] Pp. 126-7. I have quoted Carrick at some length, because he was destined to play an important part in the medical life of Bristol. In 1834, when he was senior physician at the Infirmary, he was President of the Bristol Meeting of the Provincial Medical and Surgical Association, now the British Medical Association. His address received high praise, and in the second volume of the *Transactions*, in conjunction with Dr. Symonds, he published a contribution on "The Medical Topography of Bristol." Carrick, who lived in Clifton Grove for many years, died in 1837.

sending patients in the last stage of consumption from long distances to the Hotwells. He knew "of one consumptive patient, from Scotland, who expired just as the carriage which brought him reached the door of his lodgings; of another who died the morning after his arrival; of five or six who died within the week; and of several more who did not live to reach the end of their journey."[1] Fatalities of this kind were so many in one set of houses that it received the unenviable title of "Death Row." Notwithstanding the great advantages offered to visitors, Carrick thought that there should be

7.—*One suggestion for a bridge from St. Vincent's Rocks to Leigh.*[2]

[1] P. 71.

[2] The original print, in addition to a "Ground Plan and Road," has a description of the details of the scheme. Mr. John Tremayne Lane, the City Treasurer, very kindly lent me a copy of the print from which I have taken the following:—

"*Dimensions*—The Great Arch, 220 ft. high, 180 ft. wide; Base, 400 ft. long, 140 ft. wide; Road on Top, 700 ft. long, 50 ft. wide; Each Story 40 ft. high; Gallery, 6 ft. wide.

*Contents*—No. 1, A Light House. 2, A Toll House. 3, A Chapel, called St. Vincent's. 4, 5, 6, Publick Granaries, and Corn Exchange for Foreign Grain. 7, Coal Wharf and General Market. 8, A Stone Wharf and Water Mill. 9, 10, Manufactories for Cotton, Wool, &c. 11, A Marine School. 12, A Museum, Library, and Subscription Room. 13, 14, Engine Rooms. 15, Vertical Windmills. 16, Twenty Houses in the Scite of the Bridge. 17, Various Recesses for Out Offices, Stabling, &c. 18, Clock Turret and Stair Case. 19, Watch House and Bellfry."

I presume that the raised structures near the Engine Rooms are intended for Clock Turret and Watch House on each side of the river. The numbers 18 and 19 are not in the print.

further improvements. He suggested (1) a public garden near the Mall; (2) a commodious set of baths; (3) more houses under the hill of a proper size for single families; (4) a new road from Clifton to the Hotwells; (5) a bridge over the Avon, as he did not expect ever to see erected the colossal bridge which was to have connected St. Vincent's Rocks. The picture on the previous page shows one design for a "colossal" bridge which was put forth in Carrick's time, and may be that to which he alludes.

*(To be continued.)*

---

NOTES ON THE ILLUSTRATIONS. (See also pp. 25, 26.)

3 (Inset). The use of the hay-mow is explained by the following remarks of Owen (*op. cit.*, pp. 161-2): "I would by all means advise those gentlemen who ride on horseback to the Hot-well, as many do, to put up their horses in the stable over-against the beginning of the walk, for which they pay only one penny a time for each horse, and not to tie them up to the rails while they drink the waters. This last is a very common practice; you see a whole range of them together; and the consequence is, that almost every morning one or other of them is obliged, by the backing of coaches or chariots against them, either to break his bridle, or to pull down part of the rails. By this penny-saving scheme of the owner, the horse is greatly frightned, and often receives an injury, while his master is sitting in the Pump-room, or is got to the coffee-house adjoining to it to read the news papers, and knows nothing of the matter. Many a good horse has been hurt by this bad practice, and the company, who are in the walk, are frequently alarmed by it." The building near the hay-mow appears more distinctly as a stable in the first of the illustrations.

(p. 20). "Those whose affairs will not permit them to leave *London*, and whose constitutions require its assistance, have it at five-pence the bottle, if they write to Mr. *Barratt*, the master of the Hot-well, for a hamper of it to come by sea." (Owen, *op. cit.*, p. 132).

5 (p. 21). This view appears on Roque's 1750 and 1759 plans of Bristol. It was from the plate of the later issue, the lettering of which differs slightly from that of the earlier one, that Barrett's view was printed.

7 (p. 151). Many schemes for a high bridge across the Avon were before the public at the end of the eighteenth and the beginning of the nineteenth century. This illustration, bearing on it the date of 1793, represents a suggestion from the designer of the 1768 Bristol Bridge. It comes from *Bristol: Past and Present* (III. 316).

## Figures and Tables

**7. f1:**